# Gardner-Diamond Syndrome: A Comprehensive Review of Pathophysiology, Diagnosis, and Treatment

**DOI:** 10.1192/j.eurpsy.2025.1235

**Published:** 2025-08-26

**Authors:** H. J. Gomes, R. A. Moreira, J. P. Correia, J. R. Gomes

**Affiliations:** 1 Departamento de Psiquiatria e Saúde Mental, Unidade Local de Saúde do Nordeste, Bragança, Portugal

## Abstract

**Introduction:**

Gardner-Diamond Syndrome (GDS), also known as psychogenic purpura, is an uncommon psychosomatic disorder characterized by painful, ecchymotic, purpuric lesions that typically appear after a period of stress, surgical operations or hard physical work, predominantly affecting women. The pathophysiology of GDS is still poorly understood, and diagnosis is often challenging due to its overlap with other hematological, dermatological, or psychosomatic disorders. Appropriate diagnosis is essential for symptom management and patient risk reduction.

**Objectives:**

This review aims to synthesize the current knowledge on GDS, encompassing its etiology, clinical presentation, diagnostic criteria, and treatment approaches while identifying areas for future research.

**Methods:**

We performed a narrative literature review by searching PubMed, Google Scholar, and ScienceDirect articles written in English. Relevant studies, case reports, and reviews published from 1955 to 2023 were included.

**Results:**

Gardner-Diamond Syndrome predominantly affects middle-aged women, but the literature has also published reports concerning men and children. It is often associated with psychological stress, anxiety, or depression. Many authors suggest the presence of histrionic personality traits and a tendency toward somatic reactions in affected individuals. In 1955, Frank Gardner and Louis Diamond identified and described Gardner-Diamond Syndrome after observing four women who experienced recurrent bruising, accompanied by localized pain, erythema, and swelling, following minimal or no trauma. The spontaneous bruising observed in GDS lacks a hematological cause, with most studies suggesting a psychosomatic origin. The diagnosis is made with a detailed medical and psychiatric history, physical examination, laboratory examination, and exclusion of other possible causes. Psychological therapies, such as cognitive-behavioral therapy, alongside pharmacological treatments, have shown variable success in symptom management. However, most patients experience long-term cycles of remission and relapse, and a standardized treatment protocol has yet to be established.

**Conclusions:**

Gardner-Diamond Syndrome represents a complex interaction of psychological and somatic factors, highlighting the importance of a multidisciplinary approach to both diagnosis and treatment. Diagnostic challenges persist due to the absence of definitive biomarkers and reliance on exclusionary criteria. Also, GDS lacks treatment options. While psychological interventions play a central role in management, further research is needed to clarify the underlying pathophysiology and improve therapeutic outcomes. Most patients benefit from a combination of cognitive behavioral therapy and antidepressant treatment. Increasing clinician awareness may help reduce diagnostic delays and improve the quality of life for patients.

**Disclosure of Interest:**

None Declared

